# Genoprotective Properties of *Cedrus atlantica* Essential Oil: Evidence from the Comet Assay

**DOI:** 10.3390/ijms27073268

**Published:** 2026-04-03

**Authors:** Sara Diogo Gonçalves, Cristiano Silva, Raquel Garcia Diogo, Fabiana Chyczij, Verónica Esteves, Natasha Miranda, Volodymyr V. Tkach, Isabel Gaivão, Ana Caramelo

**Affiliations:** 1 Clinical Academic Center of Trás-os-Montes and Alto Douro (CACTMAD), University of Trás-os-Montes and Alto Douro (UTAD), 5000-801 Vila Real, Portugal; 2 Animal and Veterinary Research Center CECAV and Department of Genetics and Biotechnology, School of Life and Environmental Sciences, UTAD, 5000-801 Vila Real, Portugal; 3 Serviço de Medicina Dentária, Hospital da Luz de Vila Real, 5000-657 Vila Real, Portugal; 4 Unidade de Saúde Familiar Fénix, Unidade Local de Saúde de Trás-os-Montes e Alto Douro (ULSTMAD), 5000-577 Vila Real, Portugal; 5 Unidade de Cuidados na Comunidade de Alijó, Unidade Local de Saúde de Trás-os-Montes e Alto Douro (ULSTMAD), 5070-021 Alijó, Portugal; 6 School of Health, UTAD, 5000-801 Vila Real, Portugal; 7 RISE@UTAD-Health Research Network, Faculty of Medicine, University of Porto (UP), 4200-319 Porto, Portugal

**Keywords:** antigenotoxicity, *Cedrus atlantica*, Comet assay, essential oil, oxidative DNA damage, sesquiterpenes

## Abstract

Oxidative stress is one of the major contributors to DNA damage and genomic instability, emphasizing the importance of identifying natural compounds with antioxidant genoprotective potential. *Cedrus atlantica* essential oil (EO) has been widely reported to possess antioxidant properties and potential genoprotective effects due to the presence of a cohort of antioxidant compounds, including polyphenols and terpenes. Nevertheless, its effects on DNA integrity remain poorly understood. The present study aimed to evaluate the genotoxic and antigenotoxic effects of *C. atlantica* EO in human peripheral blood mononuclear cells (PBMCs) using the alkaline Comet assay. PBMCs were exposed to increasing concentrations of the EO (0.2–3% *w*/*v*) under basal conditions and in the presence of hydrogen peroxide (H_2_O_2_, 25 µM) as an oxidative DNA-damaging agent. Genetic damage was quantified by visual score, and arbitrary units were converted into a percentage of DNA in the comet tail. The EO was characterized by gas chromatography–mass spectrometry. The results showed that *C. atlantica* EO did not induce detectable genotoxic effects under the experimental conditions and within the tested concentration range (0.2–3% *w*/*v*). H_2_O_2_ exposure markedly increased DNA strand breaks, whereas co-treatment with the EO significantly attenuated H_2_O_2_-induced oxidative DNA damage, particularly at intermediate concentrations. The chemical characterization analysis revealed a sesquiterpene-rich profile dominated by cedrene- and himachalene-type compounds. Overall, these findings indicate that *C. atlantica* EO exerts antigenotoxic effects against oxidative DNA damage, supporting its genoprotective potential in moderate concentrations.

## 1. Introduction

Nowadays, genomic integrity is continuously challenged by endogenous and exogenous sources of DNA damage, including those provoked by oxidative stress. Reactive oxygen species, generated during normal cellular metabolism or from environmental exposure to xenobiotics, can induce DNA damage, leading to base modifications and chromosomal instability, and thereby causing aging, cancer development, and a wide range of chronic diseases [1,2]. Consequently, increasing attention has been directed toward natural compounds that protect DNA from oxidative damage and support cellular homeostasis.

Plant-derived essential oils have emerged as promising sources of bioactive molecules with antioxidant, anti-inflammatory, and cytoprotective properties [3,4]. However, despite their widespread use in traditional medicine, cosmetics, and aromatherapy, essential oils represent chemically complex mixtures whose biological effects on DNA integrity remain insufficiently characterized [5]. Notably, essential oils may exert dual effects, acting as protective agents at specific concentrations while inducing oxidative or genotoxic effects at others, leading to ongoing debate over their safety and dose-dependent behavior [6,7,8]. This duality highlights the importance of systematic genotoxic and antigenotoxic assessments using validated biological models.

**Figure 1:**
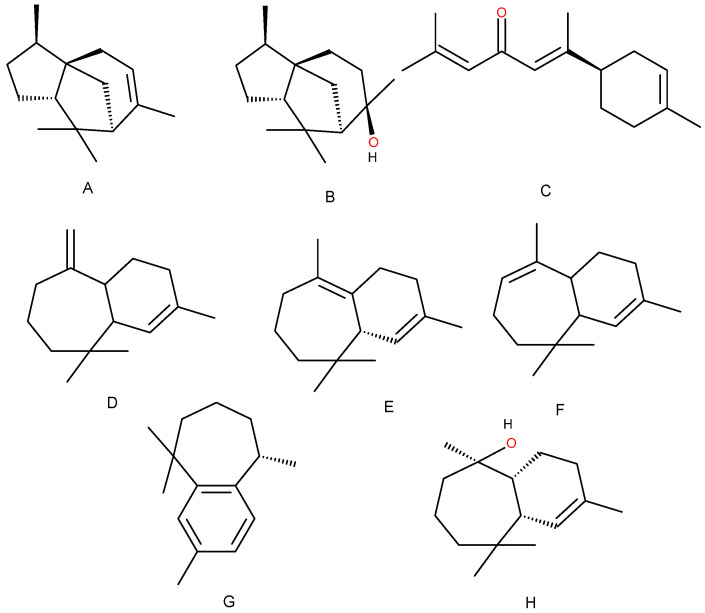
*C. atlantica* terpenes: cedrene (**A**), cedrol (**B**), atlantone (**C**), α-himachalene (**D**), β-himachalene (**E**), γ-himachalene (**F**), ar-himachalene (**G**), and himahalol (**H**). Oxygen atoms are shown in red.

*C. atlantica* essential oil (EO) is traditionally obtained from the wood of the cedar tree and is characterized by a high content of sesquiterpene hydrocarbons, particularly cedrene and himachalene derivatives, including alcohols, aldehydes, and ketones (Figure 1) [9,10]. The structural backbone of most constituents consists of bi- and tricyclic cedrane-type frameworks, biosynthetically formed from isoprene units according to the classical “head-to-tail” rule [9,11,12,13].

The main hydrocarbon components are α- and β-cedrene, which belong to the class of unsaturated bicyclic sesquiterpenes. These molecules contain one or more alkenyl moieties, which confer chemical reactivity toward radical addition reactions and autoxidation. Sesquiterpene alcohols, such as cedrol, and ketones, including atlantone mainly represent oxygenated constituents. In these molecules, functional groups such as hydroxyl (–OH) and carbonyl (C=O) play a central role in determining chemical reactivity and interaction with reactive species.

In addition to its well-characterized sesquiterpene-rich essential oil, *C. atlantica* has been shown to contain a diverse array of polyphenolic compounds in its organic extracts, which contribute substantially to its antioxidant profile [14,15,16]. Recent phytochemical investigations using HPLC-DAD-MS analysis of ethanolic needle extracts of *C. atlantica* revealed the presence of at least 16 phenolic constituents, including flavonoids and phenolic acids such as the flavone glycoside apigenin-7-O-glucoside, the flavanol catechin, and derivatives of ellagic acid (e.g., ellagic acid-deoxyhexose), among other phenolic components. These compounds are widely recognized for their free radical-scavenging and reducing abilities, mechanisms central to antioxidant activity in plant extracts and distinct from the terpene-mediated radical quenching characteristic of the essential oil fraction.

**Figure 2:**
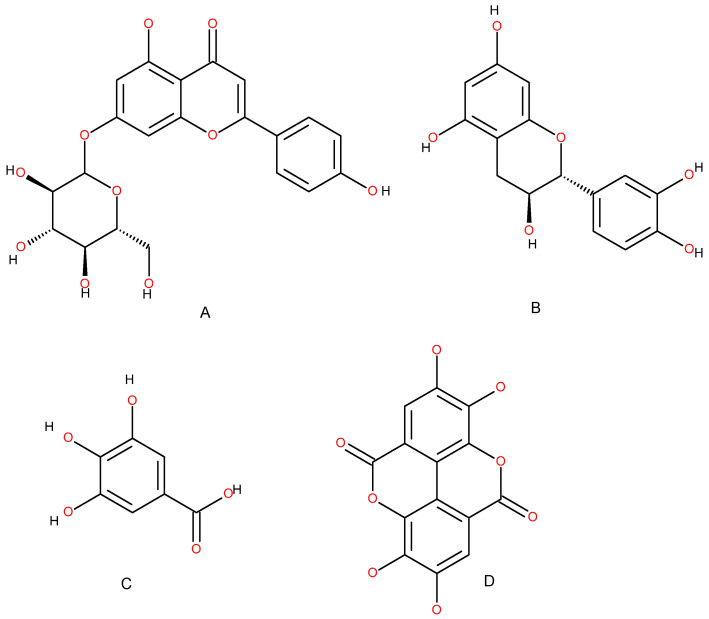
*C. atlantica* main polyphenolic backbone molecules: apigenin-7-O-glycoside (**A**), catechine (**B**), gallic acid (**C**), and ellagic acid (**D**). Oxygen atoms are shown in red.

The identification of these polyphenols—including apigenin-7-O-glucoside, a glycosylated flavone, and catechin, a flavanol with multiple hydroxyl substituents—supports the hypothesis that non-volatile phenolic metabolites contribute to the biological activity of *C. atlantica* beyond volatile terpenoids. Ellagic acid derivatives detected in the extract, such as ellagic acid-linked hexose conjugates, are representative of phenolic acid classes known for strong antioxidant and metal-chelating properties in vitro. The combined presence of these molecules suggests that the antioxidant potential of *C. atlantica* extracts arises from a synergistic interplay between flavonoid glycosides, flavan-3-ols such as catechin, and hydrolyzable tannin-like ellagic acid derivatives, each capable of donating electrons or hydrogen atoms to neutralize reactive oxygen species. This polyphenolic profile supports the growing interest in *C. atlantica* as a source of bioactive natural antioxidants with potential applications in pharmaceutical, cosmetic, and nutraceutical formulations (Figure 2).

Despite the presence of polyphenolic components, the antioxidant activity of *C. atlantica* essential oil is not primarily based on hydrogen atom donation, but rather on mechanisms of direct radical scavenging and stabilization of lipophilic radical species. Despite this, the double bonds present in sesquiterpenes are capable of reacting with peroxyl radicals (ROO·), thereby interrupting the chain reactions involved in lipid peroxidation. Oxygenated sesquiterpenes further contribute by facilitating delocalization of the unpaired electron, increasing the stability of the resulting radical intermediates. However, these polyphenolic constituents are typically associated with non-volatile extracts and are not expected to be present in significant amounts in the essential oil fraction.

From a chemical standpoint, the synergistic interactions among individual components are particularly important, as the overall antioxidant activity of the essential oil exceeds the sum of the effects of isolated constituents. The absence of significant amounts of phenolic structures results in moderate antioxidant capacity values in standard assays (such as DPPH and ABTS), while simultaneously conferring enhanced oxidative stability to the oil itself and improved performance in hydrophobic environments.

Thus, *C. atlantica* essential oil can be regarded as a lipophilic terpene-based antioxidant complex, whose activity is determined by the structural features of sesquiterpenes, the presence of unsaturated bonds and oxygenated functional groups, as well as by pronounced multicomponent synergistic effects.

*C. atlantica* (Atlas cedar) essential oil is a complex multicomponent mixture of volatile organic compounds, predominantly composed of sesquiterpenes and their oxygenated derivatives. According to gas chromatography–mass spectrometry (GC–MS) analyses, the major constituents of the oil include α-cedrene, β-cedrene, atlantone, cedrol, as well as various isomers of cedrene-derived alcohols and ketones. In contrast to many other coniferous species, the proportion of monoterpenes in *C. atlantica* essential oil is generally low [14,15,16,17,18,19].

The predominance of sesquiterpene compounds contributes to the essential oil’s high chemical stability and resistance to oxidative degradation. Oxygenated sesquiterpenes, particularly cedrol and atlantone, play a crucial role in shaping the oil’s biological activity, including its antioxidant potential.

The antioxidant activity of *C. atlantica* essential oil is demonstrated by its ability to neutralize free radicals and inhibit lipid peroxidation. Results from standard in vitro assays (DPPH, ABTS, and FRAP) indicate that the oil exhibits moderate yet consistent antioxidant activity. This effect is not associated with a high phenolic content but rather with the synergistic action of terpene constituents. Compared to phenol-rich essential oils, the antioxidant effect of *C. atlantica* is more prolonged and less sensitive to variations in oxidative stress conditions.

It has been demonstrated that the antioxidant properties of the essential oil are significantly influenced by the plant material’s geographical origin, distillation parameters, and chemotype, leading to variability in the quantitative composition of major components. Nevertheless, the general profile of biological activity remains consistent, supporting the potential application of *C. atlantica* essential oil as a natural antioxidant agent in pharmaceutical, cosmetic, and biotechnological formulations.

Previous studies have reported antioxidant, anti-inflammatory, antimicrobial, and cytoprotective activities associated with *C. atlantica* EO and its major constituents [19,20]. Sesquiterpenes, due to their lipophilic nature and relative chemical stability, have been suggested to interact with cellular membranes and modulate oxidative processes, potentially protecting biomolecules, including DNA [21]. Nevertheless, direct experimental evidence regarding the genotoxic safety and antigenotoxic potential of *C. atlantica* EO in human cells remains limited.

The alkaline Comet assay is a sensitive and widely accepted method for detecting DNA strand breaks and alkali-labile sites at the single-cell level and is extensively used for evaluating both genotoxic and antigenotoxic effects of natural products [22,23].

In this context, the present study aimed to evaluate the genotoxic and antigenotoxic effects of *C. atlantica* EO in human PBMCs using the Comet assay, both under basal conditions and following oxidative challenge with hydrogen peroxide. In parallel, the chemical composition of the essential oil was characterized by gas chromatography–mass spectrometry to ensure reproducibility and to support the interpretation of the biological findings.

## 2. Results

### 2.1. Chemical Characterization

The chemical composition of the essential oil was determined from the manufacturer’s certificate of analysis (Pranarôm, Insula Portugal). The analysis was performed by gas chromatography–mass spectrometry (GC–MS) using an Agilent system equipped with a VF-WAX column, according to the conditions specified in the supplier documentation.

The chromatographic analysis revealed a complex volatile profile characteristic of *C. atlantica* essential oil (Table 1). The oil was predominantly composed of sesquiterpene hydrocarbons and oxygenated sesquiterpenes. The major constituents identified were β-himachalene (38.63%), α-himachalene (15.18%), and γ-himachalene (9.27%), which together accounted for a substantial proportion of the total oil composition. Other notable components included E-α-atlantone (3.82%), δ-cadinene (2.38%), α-dehydro-ar-himachalene (1.66%), and γ-dehydro-ar-himachalene (1.62%).

The predominance of himachalene-type sesquiterpenes confirms the characteristic chemical fingerprint of *C. atlantica* essential oil and supports its reported antioxidant and cytoprotective potential [24,25]. Major constituents such as β-himachalene, α-himachalene, and γ-himachalene have been associated with free radical-scavenging activity and membrane-protective effects, which may contribute to the antigenotoxic responses observed in Section 3.2 [9,26]. In addition, minor components, including atlantones and δ-cadinene, although present at lower levels, are pharmacologically relevant due to their documented anti-inflammatory and antioxidant properties, potentially acting synergistically with the major sesquiterpenes [27,28].

### 2.2. Antigenotoxic Evaluation Using the Comet Assay

The genotoxic and antigenotoxic effects of *C. atlantica* EO were assessed in human PBMCs using the Comet assay under both unchallenged conditions and following H_2_O_2_-induced oxidative stress. DNA damage was quantified by visual score, converted into %DNA in tail used as indicator of basal DNA damage (single and double strand breaks and alkali labile sites) (Table 2). No morphological evidence of cytotoxicity, such as extensive DNA fragmentation or ‘hedgehog’ comets, was observed.

**Figure 3:**
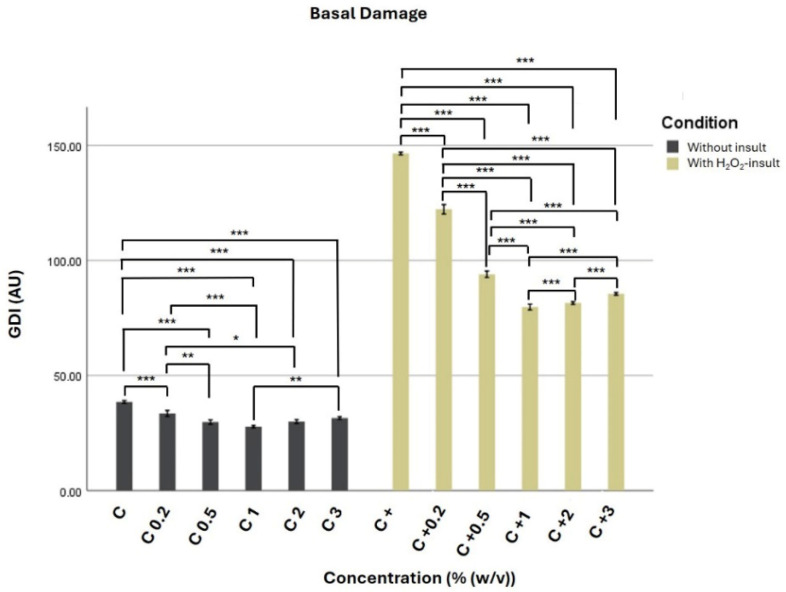
Genotoxic and antigenotoxic effects of *C. atlantica* EO in PBMCs were evaluated using the Comet assay. Bars show the mean Genetic Damage Index (GDI, arbitrary units) ± standard error. C (control) corresponds to cells treated with PBS only; C 0.2–C 3 indicate cells treated with cedarwood EO at 0.2%, 0.5%, 1%, 2%, and 3% (*w*/*v*) in the absence of a genotoxic challenge; C+ represents the positive control (PBS + H_2_O_2_, 25 µM); and C+0.2–C+3 correspond to cells co-treated with cedarwood EO (0.2–3%) and H_2_O_2_. Dark gray bars denote unchallenged groups (PBS ± EO), whereas golden bars represent H_2_O_2_-challenged groups (PBS ± EO). Asterisks indicate statistically significant differences relative to the respective control (* *p* < 0.05; ** *p* < 0.01; *** *p* < 0.001).

Under unchallenged conditions, treatment with Cedarwood EO at concentrations ranging from 0.2% to 3% (*w*/*v*) did not induce genotoxic effects and was associated with a modest but consistent reduction in basal DNA damage compared with the negative control (PBS only). The lowest %DNA in tail value was observed at 1% EO (6.938% DNA in tail), corresponding to an approximate 28% decrease relative to control cells (38.5 AU). Treatments at 0.5%, 2%, and 3% EO also resulted in reduced %DNA in tail values (7.434–7.875% DNA in tail), indicating that no genotoxic effects were detected at the tested concentrations under the conditions of the Comet assay (Figure 3).

Exposure of PBMCs to H_2_O_2_ (25 µM) led to a marked increase in DNA damage, confirming the effectiveness of the oxidative challenge (%DNA in tail = 36.67). Co-treatment with *C. atlantica* EO significantly attenuated H_2_O_2_-induced DNA damage in a non-linear, concentration-dependent manner, with a peak antigenotoxic effect at 1% (*w*/*v*) and no further improvement at higher concentrations. Although all concentrations significantly reduced DNA damage compared with the positive control, the differences between intermediate and higher concentrations were less pronounced, suggesting a plateau effect. The strongest antigenotoxic effect was observed at 1% EO, where the %DNA in tail was reduced to 19.938%, approximately 46% lower than with H_2_O_2_ alone. Similar protective effects were observed at 0.5%, 2%, and 3% EO, with %DNA in tail values ranging from 20.375% to 23.5%, all of which remained significantly lower than those in the positive control.

The percentage of DNA in the comet tail followed a pattern consistent with %DNA in tail results, showing an apparent reduction in strand break intensity in EO-treated groups relative to H_2_O_2_-exposed cells. Although the lowest EO concentration (0.2%) provided limited protection against oxidative damage, intermediate concentrations (0.5–1%) were more effective, suggesting an optimal concentration range for antigenotoxic activity.

Therefore, these findings indicate the absence of detectable genotoxic effects within the tested concentration range and experimental conditions. Moreover, the essential oil exerts a significant genoprotective effect against oxidative DNA damage. Unlike the hormetic pattern previously observed with sage EO, Cedarwood EO displayed a more pronounced antigenotoxic effect at intermediate concentrations, underscoring differences in dose–response behavior, related to its sesquiterpene-rich chemical composition [29]. The phenolic fraction, although minor, also plays an important role in the antigenotoxic behavior of *C. atlantica* EO.

## 3. Discussion

The present study demonstrates that *C. atlantica* EO does not induce detectable genotoxic effects under the experimental conditions employed. Using the alkaline Comet assay, a sensitive and widely accepted method for detecting DNA strand breaks and alkali-labile sites, we showed that exposure to Cedarwood EO alone did not increase basal DNA damage and, in contrast, was associated with a modest reduction in endogenous genomic instability. These findings support the genoprotective profile of *C. atlantica* EO and extend previous reports on the cytoprotective and antioxidant properties of cedarwood-derived products.

Chemical characterization confirmed that the EO used in this study is dominated by sesquiterpene hydrocarbons, particularly β-himachalene, α-himachalene, and γ-himachalene, which together accounted for more than 60% of the total composition. This chemical fingerprint is consistent with authentic *C. atlantica* oils from Moroccan origin and aligns with previous studies reporting antioxidant, membrane-stabilizing, and anti-inflammatory activities associated with himachalene-type compounds [24]. Sesquiterpenes are known to interact with lipid bilayers and modulate oxidative processes, which may help preserve the integrity of cellular and nuclear membranes under stress conditions [30].

Under unchallenged conditions, all tested concentrations of Cedarwood EO (0.2–3% *w*/*v*) were non-genotoxic and resulted in a slightly lower %DNA in tail values than the negative control. The most pronounced reduction in basal DNA damage was observed at 1% EO, suggesting an intrinsic capacity of the oil to counteract endogenous oxidative stress. Basal DNA damage in PBMCs is primarily attributed to reactive oxygen species generated during normal cellular metabolism, and the observed decrease may reflect free radical-scavenging activity or enhancement of cellular antioxidant defenses by EO constituents [31,32].

The antigenotoxic potential of *C. atlantica* EO was particularly evident following oxidative challenge with H_2_O_2_. As expected, exposure to H_2_O_2_ resulted in a substantial increase in DNA strand breaks, validating the effectiveness of the experimental model. Co-treatment with Cedarwood EO significantly reduced H_2_O_2_-induced DNA damage at all concentrations tested, with the most potent protective effect observed at intermediate concentrations (0.5–1% *w*/*v*). At 1% EO, DNA damage was reduced by nearly half compared with the positive control, highlighting a robust protective response against oxidative insult.

This concentration-dependent pattern suggests that Cedarwood EO exerts optimal antigenotoxic activity within a defined dose range, rather than displaying a strictly linear response. Such behavior is commonly observed in complex natural mixtures, where synergistic interactions among constituents may enhance biological activity at certain concentrations, only to plateau at higher doses. The absence of increased DNA damage at the highest concentrations tested further supports the safety of Cedarwood EO in this in vitro model and distinguishes it from essential oils that exhibit biphasic or hormetic genotoxic profiles [29].

The reduction in the percentage of DNA in the comet tail indicates that Cedarwood EO effectively limits strand break formation and/or facilitates rapid protection of DNA from oxidative attack. Mechanistically, this effect may be attributed to direct scavenging of reactive oxygen species, chelation of transition metals involved in Fenton reactions, or stabilization of chromatin structure [31,32]. In addition, minor constituents such as atlantones and δ-cadinene, though present in lower amounts, may contribute additively or synergistically to the observed antigenotoxic effects through their reported antioxidant and anti-inflammatory actions [33,34]. In cellular and acellular systems, catechin and apigenin derivatives have been shown to decrease strand breaks, micronuclei formation, and oxidative base modifications, supporting their classification as antigenotoxic phytochemicals acting primarily through redox modulation and protection of genomic integrity [35,36,37,38].

The antigenotoxic effects observed in this study are more plausibly attributed to the sesquiterpene-rich composition of *C. atlantica* essential oil, as confirmed by the chemical profile. Major constituents such as himachalene isomers and oxygenated sesquiterpenes (e.g., atlantones) have been reported to exhibit antioxidant and membrane-stabilizing properties [35]. Due to their lipophilic nature, these compounds can interact with cellular membranes and may reduce oxidative DNA damage by limiting lipid peroxidation and modulating the propagation of reactive oxygen species [39].

Given the absence of direct evidence for non-volatile phenolic compounds in the essential oil, their contribution to the observed antigenotoxic effects cannot be assumed in this study. Nevertheless, its impact (direct and indirect) on the EO antigenotoxicity may be investigated in future research.

Taken together, these findings emphasize that the biological effects of *C. atlantica* cannot be interpreted solely on the basis of qualitative composition, but must be evaluated within a quantitative and mechanistic framework. The antioxidant and antigenotoxic properties of terpenoid constituents are beneficial only within defined concentration ranges, beyond which adverse prooxidant and genotoxic effects may emerge. This duality has important implications for the safe and effective use of *C. atlantica*–derived products, particularly in pharmaceutical, cosmetic, and biotechnological applications, where controlled formulation and rigorous dose–response assessment are essential [40].

Unlike the hormetic (biphasic) dose–response pattern previously reported for sage essential oil in a comparable Comet assay model [29], *C. atlantica* EO exhibited a more pronounced antigenotoxic effect at intermediate concentrations, suggesting that differences in chemical composition—particularly the predominance of sesquiterpenes—may influence biological response profiles. The predominance of sesquiterpenes in *C. atlantica*, as opposed to monoterpene-rich oils, may underlie these differences, as sesquiterpenes generally exhibit lower volatility, higher lipophilicity, and prolonged interactions with cellular membranes [22].

Taken together, these findings contribute to the growing body of evidence supporting the biological relevance of *C. atlantica* essential oil in modulating oxidative stress and protecting genomic integrity. Further studies are required to confirm these effects under more complex biological conditions.

### 3.1. Limitations

#### 3.1.1. Chemical Composition Variability

Although the chemical profile of the essential oil used in this study was characterized and shown to be consistent with reported *C. atlantica* chemotypes, essential oils are inherently variable natural products. Factors such as geographical origin, harvesting conditions, plant age, and extraction methods can influence chemical composition, potentially affecting biological activity. Therefore, the observed effects may not fully represent all commercial or wild-sourced Cedarwood essential oils.

#### 3.1.2. Lack of Independent GC–MS Verification

The chemical characterization of the essential oil was based solely on the manufacturer’s certificate of analysis. No independent GC–MS analysis was performed by the authors to verify the composition. Although the reported profile is consistent with known *C. atlantica* chemotypes, reliance on supplier data may limit analytical robustness and reproducibility. Independent verification would strengthen confidence in the chemical characterization and its correlation with the observed biological effects.

#### 3.1.3. In Vitro Experimental Model

This study is limited by the use of an in vitro PBMC model, which does not fully capture in vivo complexity, including metabolism, bioavailability, and systemic responses. Factors such as metabolism, bioavailability, tissue distribution, and systemic antioxidant responses are not accounted for in this experimental model and may influence the genoprotective effects of *C. atlantica* EO under physiological conditions.

#### 3.1.4. Lack of Mechanistic Insight at the Molecular Level

The Comet assay provides sensitive detection of DNA strand breaks but does not identify the molecular mechanisms underlying the observed protective effects. This study did not assess oxidative stress biomarkers, antioxidant enzyme activity, DNA repair pathways, or signaling cascades involved in the cellular response to oxidative damage. As a result, the precise biological processes responsible for the antigenotoxic activity of cedarwood EO remain unresolved.

#### 3.1.5. Limited Range of Concentrations and Exposure Times

Only a defined range of EO concentrations (0.2–3% *w*/*v*) and a single exposure duration were evaluated. While these conditions were selected based on previous Comet assay studies to ensure non-cytotoxicity, they may not fully reflect the dose–response relationship or the temporal dynamics of DNA damage and protection. Different concentrations or exposure periods could yield distinct genotoxic or antigenotoxic profiles.

#### 3.1.6. Lack of Cytotoxicity Assessment

This study did not include a direct cytotoxicity evaluation (e.g., cell viability or membrane integrity assays). Although the selected concentrations were based on previous studies and no morphological signs of cytotoxicity were observed in the Comet assay, the absence of quantitative cytotoxicity data limits the interpretation of the results. Future studies should include complementary assays to confirm the safety profile of *C. atlantica* EO.

### 3.2. Future Research

Future studies should expand the evaluation of *C. atlantica* EO by including PBMCs from multiple donors of different ages, sexes, and health backgrounds in order to assess inter-individual variability and improve the translational relevance of the findings. Validation of the observed genoprotective effects in in vivo models would further clarify the biological significance of Cedarwood EO under physiological and pathophysiological conditions, particularly in the context of oxidative stress-related disorders.

Additional research should explore a broader range of concentrations and exposure times, as well as repeated or chronic exposure scenarios, to better characterize dose–response relationships and temporal patterns of genotoxicity and antigenotoxicity. Comparative studies with other oxidative stressors and known genotoxic agents would also help define the specificity and robustness of the protective effects observed.

Mechanistic investigations are warranted to elucidate the molecular pathways underlying the antigenotoxic activity of Cedarwood EO. These may include assessments of intracellular reactive oxygen species levels, antioxidant enzyme activity, lipid peroxidation, DNA repair capacity, and the modulation of redox-sensitive signaling pathways. Fractionation of the essential oil and testing of individual major and minor constituents could further clarify their respective contributions and potential synergistic interactions.

Finally, future work should prioritize comprehensive chemical standardization, including independent GC–MS analyses and batch-to-batch comparisons, to ensure reproducibility and facilitate cross-study comparisons. Such efforts will be essential for advancing Cedarwood essential oil toward potential biomedical or preventive applications.

## 4. Materials and Methods

### 4.1. Cells

Peripheral blood samples were collected from a healthy 37-year-old female volunteer. The participant was a non-smoker, did not consume alcohol, had no known medical conditions, and had not taken any medication during the six months preceding the study. The use of a single donor was intentionally adopted to minimize inter-individual variability and ensure that all experimental conditions were evaluated under identical biological backgrounds, allowing a direct and unbiased comparison between treatments. The study was conducted in accordance with the Declaration of Helsinki and was approved by the Ethical Committee for Health of the Local Health Unit of Trás-os-Montes and Alto Douro (CES REG. 1179).

### 4.2. Chemicals

Cedarwood essential oil (INCI: Cedrus Atlantica Oil; lot OF010263) was obtained from Pranarôm, Inula Portugal, Unipessoal, Lda. (Guilhabreu, Portugal). All other chemicals and reagents were purchased from Sigma-Aldrich Chemical Company (Madrid, Spain).

### 4.3. Lysis Solution

The lysis solution was prepared by combining 2.5 M NaCl, 0.1 M disodium EDTA, and 0.01 M Tris base in distilled water, with the pH adjusted to 10 using 10 M NaOH. Just prior to use, 1% Triton X-100 was added to complete the lysis solution.

### 4.4. Phosphate-Buffered Saline (PBS) Solution

PBS solution was prepared by dissolving 2 mM KH_2_PO_4_, 10 mM Na_2_HPO_4_, 2.7 mM KCl, and 137 mM NaCl in distilled water, using slightly less than the final target volume. The pH was carefully adjusted to 7.4 with 1 M HCl, after which additional distilled water was added to reach the final desired volume.

### 4.5. Electrophoresis Solution

The electrophoresis solution was freshly prepared daily by mixing 0.3 M NaOH and 1 mM EDTA in a flask, then gradually adding distilled water to the final volume, and adjusting the pH to 12.6. The pH was monitored with a calibrated meter and adjusted as needed by adding small amounts of NaOH or EDTA solution. This procedure was repeated until the target pH was achieved. The final solution was filtered to remove particulates and stored in a clean, labeled container.

### 4.6. Cedarwood Essential Oil Treatment

The selected concentrations of Cedarwood EO (0.2%, 0.5%, 1%, 2%, and 3% *w*/*v*) were based on previous studies to ensure non-cytotoxic conditions in Comet assay experiments [3,41], ensuring both biological relevance and non-cytotoxicity under in vitro conditions. For each concentration, two independent experiments were conducted, separated by 10 days.

For H_2_O_2_ treatment, a fresh working solution was prepared immediately prior to each experiment by diluting hydrogen peroxide in PBS to a final concentration of 25 µM. Briefly, an aliquot of stock H_2_O_2_ solution was added to 0.5 mL of PBS and gently mixed to ensure homogeneity. To prevent degradation and loss of activity, the solution was prepared immediately before use, protected from light, and kept on ice until application. This approach ensured a stable and reproducible oxidative stimulus during the assay [42].

**Figure 4:**
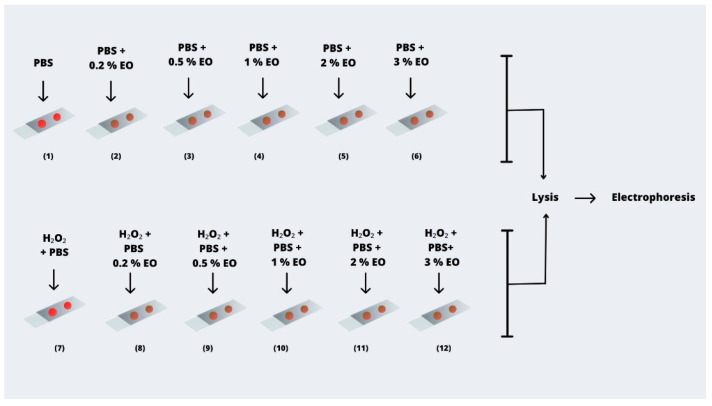
Illustration of the H_2_O_2_ treatment setup: 12 slides were prepared. Following blood collection via a finger prick, the gel matrix on each slide was treated with the designated solutions. Slide 1 contained PBS only; slides 2–6 contained PBS combined with increasing concentrations of Cedarwood EO. Slide 7 contained PBS with H_2_O_2_, and slide 8 also contained PBS with H_2_O_2_. Slides 9–12 combined PBS, H_2_O_2_, and Cedarwood EO at concentrations of 0.2%, 0.5%, 1%, 2%, and 3% *w*/*v*, respectively. All slides were processed following standard lysis and electrophoresis procedures.

The experimental setup involved 12 slides: the first contained PBS only; slides two through six contained PBS combined with increasing concentrations of Cedarwood EO (0.2%, 0.5%, 1%, 2%, or 3% *w*/*v*); the seventh contained H_2_O_2_ only; and slides eight through twelve combined H_2_O_2_ with the respective EO concentrations. A schematic overview of the experimental workflow is provided in Figure 4. No organic solvent (e.g., DMSO or ethanol) was used for essential oil preparation. The EO was directly dispersed in PBS or PBS containing H_2_O_2_ and vigorously mixed immediately prior to use to ensure adequate dispersion. This approach was adopted to avoid potential solvent-related interference with DNA damage outcomes. This procedure is consistent with previously reported Comet assay studies using essential oils [3,43].

### 4.7. Genotoxic Evaluation: Comet Assay in Human PBMCs Using H_2_O_2_

The genotoxic and antigenotoxic effects of Cedarwood EO were evaluated using an in vitro Comet assay in human PBMCs, following previously described methods [23,43]. All solutions and pre-coated slides with 1% normal-melting-point agarose were prepared the day prior. Blood samples were collected via finger prick, and 25 μL of blood was mixed with 0.8% low-melting-point agarose in PBS. Two 70 μL aliquots of this mixture were placed on each pre-coated slide and covered with coverslips to ensure even distribution. This procedure was repeated for each EO concentration tested.

Slides were incubated at 4 °C for 5 min to allow the agarose to solidify. Coverslips were then removed, and slides were immersed in Coplin jars containing the designated treatments at 37 °C for 1 h, after which they were transferred to lysis solution at 4°C.

For DNA denaturation and electrophoresis, one slide per condition was placed in the chamber without gaps, which was then filled with cold alkaline denaturing and electrophoresis buffer until the slides were fully submerged. The chamber was left for DNA denaturation for 20 min. Electrophoresis was performed in the dark at 4 °C with a current of 300 mA and a voltage of 25 V (0.8 V/cm) for 20 min.

Following electrophoresis, slides were washed sequentially in PBS (10 min, 4 °C) and distilled water (10 min, 4 °C), then air-dried. Each gel was stained with 40 μL of DAPI (4′,6-diamidine-2-phenylindol dihydrochloride) (1 μg/mL in ultra-pure water), and a coverslip was placed on it. Slides were examined using a Leica DMLS fluorescence microscope (400× magnification, Exton, PA, USA).

Each experimental condition was performed in two independent biological replicates (blood collected on separate days, 10 days apart) and two technical replicates per condition. For each slide, 50 cells were scored, yielding a total of 100 cells analyzed per experimental point. Tail intensity for each cell was scored on a scale from 0 (no tail) to 4 (almost all DNA in tail), and the final score, expressed in arbitrary units ranging from 0 to 400, was calculated using the following formula:

$$\begin{array}{c} Genetic\;Damage\;Indicator\;(GDI)\;\\ \;\;\;\;\;\;\;\;\;\;=[(\%\;nucleoid\;class\;0)\times 0)]\\ \;\;\;\;\;\;\;\;\;\;+\;[(\%\;nucleoid\;class\;1)\times 1)]\\ \;\;\;\;\;\;\;\;\;\;+\;[(\%\;nucleoid\;class\;2)\times 2)]\\ \;\;\;\;\;\;\;\;\;\;+\;[(\%\;nucleoid\;class\;3)\times 3)]\\ \;\;\;\;\;\;\;\;\;\;+\;[(\%\;nucleoid\;class\;4)\times 4)] \end{array}$$

DNA damage was expressed as the percentage of DNA in the tail, calculated by dividing the AU value by 4, providing a more standardized parameter, as described by Azqueta et al. [44].

### 4.8. Chemical Characterization

The Cedarwood EO used in this study was supplied by Pranarôm (Inula Portugal, Unipessoal, Lda., Leiria, Portugal), and its chemical profile was obtained from the certificate of analysis provided by the manufacturer (Pranarôm, Inula Portugal, Unipessoal, Lda., Leiria, Portugal). According to the supplier, the analysis was performed by gas chromatography–mass spectrometry (GC–MS) coupled with flame ionization detection (FID) using an Agilent system (Santa Clara, CA, USA) equipped with a VF-WAX column (60 m × 0.25 mm × 0.25 µm).

The reported analytical conditions included an initial temperature of 60 °C (held for 10 min), followed by a 2 °C/min increase to 250 °C, with a final hold of 15 min. Helium was used as the carrier gas at a pressure of 23 psi, and the detection limit for individual constituents was 0.01%.

No independent GC–MS analysis was performed by the authors.

### 4.9. Statistical Analysis

Data were analyzed using IBM SPSS Statistics (version 20; IBM Corp., Chicago, IL, USA). One-way analysis of variance (ANOVA) was performed, followed by Tukey’s post hoc test. Differences were considered statistically significant at *p* < 0.05.

## 5. Conclusions

This study demonstrates that *C. atlantica* EO is non-genotoxic in human PBMCs and provides significant protection against H_2_O_2_-induced oxidative DNA damage. The observed antigenotoxic effects, particularly at intermediate concentrations, are consistent with the oil’s sesquiterpene-rich composition. Overall, these findings indicate that *C. atlantica* EO exerts antigenotoxic effects against oxidative DNA damage under the tested conditions, supporting its genoprotective potential within a defined concentration range. *C. atlantica* essential oil did not induce detectable genotoxic effects and reduced oxidative DNA damage in human PBMCs under the experimental conditions and within the tested concentration range (0.2–3% *w*/*v*). However, these findings should not be extrapolated beyond these conditions. Further studies, including broader concentration ranges and direct cytotoxicity assessments, are required to establish its safety profile fully.

## Figures and Tables

**Table 1:** Major constituents of *Cedrus atlantica* essential oil determined by GC–MS.

Compound	Chemical Class	Percentage (%)
β-Himachalene	Sesquiterpene hydrocarbon	38.63
α-Himachalene	Sesquiterpene hydrocarbon	15.18
γ-Himachalene	Sesquiterpene hydrocarbon	9.27
E-α-Atlantone	Oxygenated sesquiterpene	3.82
δ-Cadinene	Sesquiterpene hydrocarbon	2.38
α-Dehydro-ar-himachalene	Sesquiterpene hydrocarbon	1.66
γ-Dehydro-ar-himachalene	Sesquiterpene hydrocarbon	1.62

Total identified compounds: 99.71%.

**Table 2:** %DNA in tail in human PBMCs exposed to different concentrations of Cedarwood EO, with and without genotoxic insult (H_2_O_2_).

Treatment	%DNA in Tail
C	9.625
C 0.2	8.375
C 0.5	7.434
C 1	6.938
C 2	7.5
C 3	7.875
C +	36.667
C +0.2	30.563
C +0.5	23.5
C +1	19.938
C +2	20.375
C +3	21.375

C: negative control (PBS); C 0.2–C 3: treatments with cedarwood EO (0.2–3%); C +: positive control (PBS + H_2_O_2_); C +0.2–C +3: treatments with cedarwood EO (0.2–3%) and H_2_O_2_.

## Data Availability

Data supporting the findings and conclusions are available upon request from the corresponding author, due to privacy or ethical restrictions.

## Abbreviations

The following abbreviations are used in this manuscript:

EO	Essential oil
GCMS	Chromatography–mass spectrometry
GDI	Genetic Damage Indicator
PBMCs	Peripheral blood mononuclear cells
PBS	Phosphate-Buffered Saline
